# Convergent validity of the Autism Spectrum Disorder Mealtime Behavior Questionnaire (ASD-MBQ) for children with autism spectrum disorder

**DOI:** 10.1371/journal.pone.0267181

**Published:** 2022-04-28

**Authors:** Kazuyo Nakaoka, Hiroyuki Tanba, Takuma Yuri, Kiyomi Tateyama, Shigeki Kurasawa

**Affiliations:** 1 Graduate School of Rehabilitation Science, Osaka Metropolitan University, Osaka, Japan; 2 Department of Occupational Therapy, Kansai University of Welfare Science, Osaka, Japan; 3 Rehabilitation Unit, Kyoto University Hospital, Kyoto, Japan; 4 Department of Occupational Therapy, Fukushima Medical University, Fukushima, Japan; Chiba Daigaku, JAPAN

## Abstract

There was a growing interest in difficulties with eating as one of the most problematic symptoms in children with autism spectrum disorder (ASD). The purpose of this study is to examine the convergent validity of the Autism Spectrum Disorder Mealtime Behavior Questionnaire (ASD-MBQ) with the Asahide’s test for social adjustment skills, the Japanese version of the short version of the sensory profile questionnaire, the Japanese version of the Social Communication Questionnaire, and the Parenting Strain Index in Japanese children with ASD. The final sample contained 294 children who were diagnosed as the ASD based on the DSM-5 criteria, the mean age of children was 10 ± 4 years (range: 3–18 years). The ASD-MBQ is a questionnaire that comprised of 42 items that are classified into five subdomains: selective eating, clumsiness/manners, interest in/concentration on eating, oral-motor function, and overeating. Spearman’s rank correlation coefficient revealed that the ASD-MBQ showed the moderate to high correlation coefficient with all four external criteria. Our results in this study supported the sufficient convergent validity in the ASD-MBQ. Therefore, the ASD-MBQ could be a useful tool for research and practice in a wide range of children with ASD aged 3 to 18.

## Introduction

There was a growing interest in difficulties with eating as one of the most problematic symptoms in children with autism spectrum disorder (ASD) [1]. For example, the association between selective eating and sensory sensitivity in children with ASD has been reported, and it is well known that selective eating negatively affects health conditions and education in daily life [2]. Furthermore, difficulties with feeding are associated with parental stress and adversely impact family functioning [3]. The Brief Autism Mealtime Behavior Inventory (BAMBI) was designed to measure mealtime behavior problems observed in children with ASD and widely used in English countries [4,5]. This validated tool let researchers and clinicians develop an intervention for mealtime behavior in children with ASD. However, behavior during eating differs among countries and cultures. Besides, there is no Japanese version to evaluate the mealtime problems in children with ASD.

To overcome these limitations, the Autism Spectrum Disorder-Mealtime Behavior Questionnaire (ASD-MBQ) has been developed to assess mealtime behavior in Japanese children [6,7]. The ASD-MBQ is comprised of 42 items that are classified into five subdomains: (1) selective eating, (2) clumsiness/manners, (3) interest in/concentration on eating, (4) oral-motor function, and (5) overeating. The ASD-MBQ is appealing for several reasons; it is applicable to a wide age range (3 to 18 years) and children with ASD diagnosed by DSM-5 criteria, as well as the items evaluated not only the symptomatic behaviors during mealtime but also the social aspect of eating such as clumsiness/manners. Items were responded to on a Likert scale by caregivers. Previous studies demonstrated that the Cronbach’s alpha coefficient was 0.930 overall and ranged from 0.781 to 0.923 among the five-subdomains [6], and that the construct (structural) validity was assessed using exploratory factor analysis and the confirmatory factor analysis [7]. Those have been shown to a model fit. However, convergent validity, which refers to the degree that the new scale is related to the other scales or measures whose constructs should theoretically be related to each other, of the ASD-MBQ remains unknown. Once the convergent validity of the ASD-MBQ is examined, we will proceed the steps to investigate the discriminate validity so that the care givers and clinicians can assess the meal-time behaviors in the Japanese children with possible ASD and provide cares with them. Therefore, the objective of this study was to examine the convergent validity of the ASD-MBQ with other measurements that have previously been validated. To examine convergent validity, we investigated the following hypothesis: the mealtime behavior in children with ASD could be associated with the severity of symptoms, sensory sensitivity, social adjustment skill, and parental stress.

## Methods

### Participants

Children, aged 3–18 years old and diagnosed with ASD, were randomly recruited from local facilities, hospitals, and organizations that serve children with ASD in Japan. Inclusion criteria for this study were as follows: (1) children diagnosed with ASD by a medical doctor, based on the DMS-V criteria (2) age between 3 and 18 years, and (3) parents/caregivers who provided their child’s age, and gender, as well as the responses of all items in the ASD-MBQ and four external criteria. The participants who incorrectly respond to all items were excluded. This study was conducted in accordance with the committee on research ethics of (# 2016–207) and performed in accordance with their ethical standards and those of the 1964 Helsinki declaration and its later amendments. Written informed consent to participate in the study was obtained from all patients.

### Measures

The ASD-MBQ was developed to capture mealtime behaviors specific to children with ASD aged 3–18 years [6,7]. The ASD-MBQ examined a series of problematic behaviors by a 42-item parent/caregiver-report questionnaire. Items were consisted of 5 sub-categories; “selective eating”, “clumsiness/manners”, “interest in/concentration on eating”, “oral-motor function”, and “overeating”. Each item asked the parent/caregiver to answer how often his/her children engage in a specific mealtime behavior with a five-point Likert scale; 1 indicates that a specific behavior was never observed within a week and 5 indicates that the behavior is always observed. Higher scores indicated the presence of more problematic behaviors (Table 1). Previous study indicated that the Cronbach’s alpha coefficient was 0.930 overall and ranged from 0.781 to 0.923 among the five-factors [6]. The structural validity was assessed using exploratory factor analysis and the confirmatory factor analysis, and those have been shown to a model fit [7].

**Table 1:** ASD-MBQ.

Selective Eating
1 The child only eats a few types of foods.
2 The child only eats certain foods/ingredients or products of certain manufacturers.
3 The child does not eat food with a strong smell.
4 The child does not eat some foods due to their appearance.
5 The child does not want tastes to be blended.
6 The child does not eat some foods due to the color.
7 Places to eat out are limited/restricted.
8 The child does not eat food that he/she has never eaten.
9 The child does not eat school meals.
10 The child eats a narrow variety of foods.
11 The child does not eat food when the shape is changed.
**Clumsiness/Manners**
12 The child eats with his/her hands, not with utensils.
13 The child walks away from the table during mealtime.
14 The child cannot wait and eats soon after served.
15 The child has difficulty handling chopsticks.
16 The child has difficulty with certain actions (opening the package or wrapping paper of food, opening the cap of a plastic bottle, etc.).
17 The child does not hold a bowl while eating.
18 The child is unable to set a meal.
19 The child has difficulty handling a spoon/fork.
20 The child plays with food or utensils.
21 The child is unable to wait until mealtime.
22 The child spills lots of food.
23 The child is unable to clear up dishes after a meal.
**Interest in/Concentration on Eating**
24 The child seems to have no appetite.
25 The child rattles a chair or table during mealtime.
26 The child is unable to concentrate on eating.
27 The child is unable to maintain a good posture during mealtime.
28 The child continues to talk using one side during mealtime.
29 The child does not seem to feel hungry.
30 The child is unable to eat while talking and listening to music.
31 The child takes a lot of time to finish a meal.
32 The child is unable to switch from a previous activity to start a meal.
**Oral-motor Function**
33 The child swallows food too quickly.
34 The child does not chew the food enough.
35 The child eats too quickly.
36 The child stuffs the mouth with food.
37 The child swallows food without chewing.
**Overeating**
38 The child is unable to control the amount of food he/she eats.
39 The child seems to not feel full.
40 The child eats all the food in front of him/her.
41 The child eats more food than do children at the same age.
42 The child eats a lot of food in addition to three meals.

The Asahide’s test was used to measure social adjustment skills in children [8]. It consists of 192 items: 57 for communication; 39 for the living; 51 for social skills; and 52 for interpersonal skills suitable for Japanese culture. Caregivers responded on a 2- or 3-point scale, and scores ranged from 0–366 were obtained. Higher scores indicated higher social adjustment skills. This is one of the most frequently used questionnaires to assess the social adjustment skills in the children with ASD. The age-range for Asahide’s test is 3 to 18 years old, namely same age-range to ASD-MBQ.

The Japanese short version of sensory profile questionnaire (SPQ) was used to assess responses to sensory stimuli and difficulties, such as sensory hypersensitivity and hyposensitivity, which can be comprehensively understood in multiple sensory regions [9,10]. This test consists of 38 items and caregivers respond with a 5-point Likert scale (1 to 5). Higher scores indicated the presence of more sensory difficulties.

The Japanese version of Social Communication Questionnaire (SCQ) was used to assess communication and interpersonal skills [11]. This measure contains 40 items. Caregivers responded to “Yes” or “No” questions. Higher scores indicated higher-level autism. Total scores were calculated, higher score indicated strong ASD symptoms. The cutoff value for ASD diagnosis in the Japanese version of SCQ is more than 7 points [12].

The Parenting Strain Index was used to evaluate the burden for parent/caregiver of children with ASD in Japan [13]. This index consists of 8 items: 4 items relating to negative feelings towards the child and 4 items relating to restrictions in the caregiver’s social activity. Caregivers responded on a 5-point Likert scale, and total scores between 0–32 were calculated. Higher scores indicated a higher childcare burden.

### Procedures

The survey including the ASD-MBQ, the Asahide’s test, the SCQ, the SPQ, and the Parenting Strain Index was mailed to participants who attends local facilities, hospitals, and organizations in Japan and who consented to participate in this study. It took about 5 to 10 min for the parents to complete the ASD-MBQ. The participants who returned the survey and completed all the items were converted to further analysis. The normality of data evaluation using Shapiro-Wilk test indicated a non-normal distribution in all data. Therefore, the convergent validity was examined using Spearman’s rank correlation coefficients between the total score in each external criterion and the total score of the ASD-MBQ, as well as the scores in five subdomains. The effect size of correlation was defined based on the Evans as follows: very weak (r < 0.2), weak (0.2 < = r < 0.4), moderate (0.4 < = r < 0.6), strong (0.6 < = r < 0.8), very strong (r > = 0.8) [14].

## Results

Participants in this study were comprised of 294 patients/caregivers who provided all demographic data and responded to all measures. The mean age of children with ASD was 10 ± 4 years (range: 3–18 years). 229 were male and, 65 were female. The SCQ score was used as the ASD score, and the mean score was 12.1 ± 7.3 (range: 0–33). In assessments for coexisting diseases, 18 children with ASD had the learning disorder, 52 had the attention deficit hyperactivity disorder, and 64 had the intellectual disability. Table 2 shows the overall demographic data. Age range of participants in this study is presented as S1 Table.

**Table 2:** Participant characteristics.

	Age	ASD-MBQ score	Asahide’s score	SPQ score	SCQ score	PSI score
Mean (SD)	10 (4)	88.9 (29.1)	192.1 (94.0)	75.6 (22.3)	12.1 (7.3)	11.6 (7.5)
Range	3 to 18	43 to 181	0 to 354	38 to 140	0 to 33	0 to 32

The mean total score of ASD-MBQ was 88.9 (range 43 to 181). The mean scores of subdomains of the ASD-MBQ were 24.1 (range 11 to 53), 26.3 (range 12 to 59), 18.0 (9 to 44), 10.9 (5 to 25), and 9.56 (5 to 24) for selective eating, clumsiness/manners, interest in/concentration on eating, oral-motor function, and overeating, respectively.

The mean score in Asahide’s test for social adjustment skills was 192.1 ± 94.0 (range 0 to 354). There was a moderate correlation between the Asahide’s test and the total score of ASD-MBQ (r = -.604, *p* < 0.001; Table 3). Subdomain analysis demonstrated that the correlation between Clumsiness/manners and the Asahide’s test was a strong relationship, while others indicated moderate to weak (Table 3).

**Table 3:** Spearman’s correlation coefficients of the ASD-MBQ and four criteria.

ASD-MBQ	Asahide’s test	SPQ	SCQ	PSI
Total Score	-.604	.741	.516	.455
Selective Eating	-.384	.583	.358	.216
Clumsiness/manners	-.704	.616	.517	.405
Interest in/concentration on Eating	-.369	.616	.276	.435
Oral-motor Function	-.230	.457	.326	.457
Overeating	-.419	.400	.441	.280

P values showed < 0.001 in all analysis.

Asahide’s test; Asahide’s test for social adjustment skills; ASD-MBQ, Autism Spectrum Disorder-Mealtime Behaviour Questionnaire; SPQ, the short Japanese version of the Sensory Profile; SCQ, the Japanese version of the Social Communication Questionnaire; PSI, Parenting Strain Index.

The mean score in the Japanese version of the SPQ was 75.6 ± 22.3 (range 38 to 140). We found a strong correlation between the short Japanese version of the SPQ and the ASD-MBQ (r = .741, *p* < 0.001). Further analysis revealed moderate correlations in subdomains with the short Japanese version of the SPQ (Table 3).

The mean score in the SCQ was 12.1 ± 7.3 (range 0 to 33). A moderate correlation between the SCQ and the ASD-MBQ was found (r = .516, *p* < 0.001). Subdomain analysis indicated moderate relationships with the SCQ (Table 3).

The mean score in the Parenting Strain Index was 11.6 ± 7.5 (range 0 to 32). There was a moderate correlation between Parenting Strain Index and the ASD-MBQ (r = .455, *p* < 0.001). There were moderate relationships with subdomains of the ASD-MBQ (Table 3).

The association with the sub-items of each scale is presented as S2 Table.

## Discussion

The objective of this study was to determine the convergent validity of the ASD-MBQ with four validated measures. This is the first study to show convergent validity between the ASD-MBQ and Asahide’s test for social adjustment skills, the SPQ, the SCQ, and Parenting Strain Index in children with ASD, ranging in age from 3 to 18 years. We developed the ASD-MBQ to capture problematic mealtime behaviors from not only the viewpoint of symptomatic behaviors but also the social communication aspect of a meal [6]. Our results in this study demonstrated the moderate to high correlation of the ASD-MBQ compared to four external criteria and supported that the ASD-MBQ could be used to assess mealtime behavior in children with ASD.

The highest correlation coefficient was found between the total score in ASD-MBQ and SPQ (r = 0.741, p < 0.001). Castro et al. investigated the correlation coefficient between the SPQ and the BAMBI scores for children with ASD and found a moderate correlation (r = -0.38, p < 0.001) in total scores [15]. This suggested that feeding problem may be related to the sensory proceeding pattern in the children with ASD [16]. Several studies reported that the eating problems are associated with an increase in parental stress [3,17]. Our results also showed a moderate relationship between the total score of ASD-MBQ and Parenting Strain Index (r = 0.454, p < 0.001), which is consistent with the findings previously reported [3,17]. Novel point of this study is to compare the mealtime behavior with the social communication and adjustment skills measured using the SCQ. Our results demonstrated that the ASD-MBQ showed moderate correlation coefficient with the SCQ (r = 0.516, p < 0.001) as well as the Asahide’s test for social adjustment skills (r = -0.604, p < 0.001). These results suggested the potential usefulness of the ASD-MBQ as an assessment for the social aspect of eating in children with ASD. In addition, as described above, our findings support that the ASD-MBQ has sufficient convergent validity to evaluate eating problems in children with ASD.

Regard to sub-category analysis, selective eating showed moderate correlations with the SPQ (r = 0.583, p < 0.001), but there was a low correlation with the other measurements (r < 0.384, p < 0.001). This aligns with previous findings that children with ASD prefer to eat specific kinds of food [18,19]. Interestingly, clumsiness/manners showed moderate to high correlations (0.405 < r < -0.704) between all criteria. These findings suggested that it is a strong point of the ASD-MBQ to be able to evaluate the social aspect of eating in the children with the ASD, as well as the clumsiness/manners will be a target for clinicians to focus on. Interest in/concentration on eating was related with the SPQ (r = 0.616, p < 0.001), as well as the PSI (r = 0.435, p < 0.001). Some studies reported that children with ASD had difficulties to remain seated during mealtime and such behavior led to parental stress [20,21], which consistent with our results. Oral-motor function showed moderate correlations between the SPQ (r = 0.457, p < 0.001), as well as the PSI (r = 0.457, p < 0.001). It is well known that oral motor function is one of the factors related to food refusal in children with ASD [22].

We found a moderate relationship between Clumsiness/manners, Interest in/concentration on Eating, and Oral-motor function with the PSI, but low with selective eating and overeating. Previous studies reported the caregiver’s burden related to children with ASD [3,17]. Our subcategory analysis revealed that the social aspect of eating and disruption of eating may be a main cause for parental burden, while selective eating and overeating were also known as the risk related to eating in children with ASD [23]. These findings found using the ASD-MBQ provide implications to improve interventions for mealtime behavior in children with ASD.

This study is not without limitations. First, there is no measure to evaluate the problematic mealtime behaviors specific to children with ASD in Japanese, except for the ASD-MBQ. Therefore, the convergent validity of ASD-MBQ with four measures was examined in this study. The BAMBI has been used to evaluate mealtime behaviors in children with ASD in English countries [5]. Gray et al. demonstrated that the mealtime behaviors of Chinese American children showed a unique pattern of mealtime behavior [24]. Future studies will need to examine the cross-cultural validity and convergent validity between the ASD-MBQ and the BAMBI. Second, the Asahide’s test was used as the measurement for social adjustment skills because there is no validated measure provided in the Japanese version, except for this measurement. It was developed based on the American Association on Intellectual and Developmental Disabilities and Vineland Adaptive Behavior Scale [25], which is a gold standard measurement to evaluate social adjustment skills. Further analysis will be conducted to investigate the convergent validity with other tools. Third, this study recruited relatively smaller number of participants in the young age population, compared to the older population. This is similar to prior study [4], because the number of diagnosis in this younger populations were less than the older population. In addition, the sample size we analyzed was excellent, based on the COSMIN checklist [number of samples is ≥ [(the number of items) × 7] and ≥100]. The number of items in the ASD-MBQ and samples in which factor analyses were performed were 42 and 294, respectively,　which met the excellent criterion. We successfully obtained responses from the full range of ages of children with ASD (3–18 years). The boy: girl ratio in our study was 3 to 4:1, which is similar to that reported in epidemiological studies [25]. Furthermore, 79 of the 294 were classified into a suspicious population with ASD diagnosis based on the cutoff value of Japanese version of the SCQ. Future studies including typically developing children will provide further evidence and implications of the ASD-MBQ. Forth, it should be noted that the responders of the questionnaires used in this study were caregivers. This might be one of a bias for the pure assessment for meal-time behaviors. Fifth, the Divergent (discriminant) validity of the ASD-MBQ is still unclear. Further study will investigate the Divergent (discriminant) validity of the ASD-MBQ from the typically developing children so that we can use the ASD-MBQ in the clinical settings. Sixth, some of the co-occurring psychiatric conditions in ASD are anxiety and depression [26]. Therefore, future study will be needed to investigate the relationship between the ASD-MBQ and anxiety and depression in children with ASD.

This study provided evidence of convergent validity of the ASD-MBQ compared with the Asahide’s test for social adjustment skills, the Japanese version of the short version of the sensory profile, the Japanese version of the Social Communication Questionnaire, and the Parenting Strain Index with the moderate to high correlation coefficient. The findings support the continued use and development of the ASD-MBQ as a useful tool for research and practice in a wide range of children with ASD aged 3 to 18. Future research is warranted to investigate the validity of the ASD-MBQ and the BAMBI in the children with ASD diagnosed by DSM-5.

## Supporting information

S1 Table Participant age distribution (age). (DOCX)

S2 Table a Spearman’s correlation coefficients of the ASD-MBQ and Asahide’s test. b Spearman’s correlation coefficients of the ASD-MBQ and SPQ. c Spearman’s correlation coefficients of the ASD-MBQ and PSI.
(DOCX)

S3 Table The means and standard deviations of the age, ASD-MBQ, Asahide’s test, SPQ and PSI. (DOCX)

S1 Data Raw data for participants (n = 294). (XLSX)
