# Supplementary material for: Convergent validity of the Autism Spectrum Disorder Mealtime Behavior Questionnaire (ASD-MBQ) for children with autism spectrum disorder
Source: PLoS One. 2022 Apr 28;17(4):e0267181. doi: 10.1371/journal.pone.0267181 (PMC9049548; doi:10.1371/journal.pone.0267181)
Supplement: S1 Table — (DOCX) [file pone.0267181.s001.docx]

**S1 Table** Participant Age Distribution (age)

| Age(years) | n | | | % |
| --- | --- | --- | --- | --- |
|  | Male | Female | Total |  |
| 3 | 4 | 2 | 6 | 2.0 |
| 4 | 14 | 3 | 17 | 5.8 |
| 5 | 14 | 6 | 20 | 6.8 |
| 6 | 26 | 9 | 35 | 11.9 |
| 7 | 25 | 8 | 33 | 11.2 |
| 8 | 19 | 9 | 28 | 9.5 |
| 9 | 12 | 0 | 12 | 4.1 |
| 10 | 20 | 4 | 24 | 8.2 |
| 11 | 13 | 2 | 15 | 5.1 |
| 12 | 13 | 4 | 17 | 5.8 |
| 13 | 13 | 3 | 16 | 5.4 |
| 14 | 15 | 2 | 17 | 5.8 |
| 15 | 13 | 2 | 15 | 5.1 |
| 16 | 11 | 5 | 16 | 5.4 |
| 17 | 11 | 3 | 14 | 4.8 |
| 18 | 6 | 3 | 9 | 3.1 |
| total | 229 | 65 | 294 | 100 |
