# Supplementary material for: Convergent validity of the Autism Spectrum Disorder Mealtime Behavior Questionnaire (ASD-MBQ) for children with autism spectrum disorder
Source: PLoS One. 2022 Apr 28;17(4):e0267181. doi: 10.1371/journal.pone.0267181 (PMC9049548; doi:10.1371/journal.pone.0267181)
Supplement: S2 Table — a Spearman’s correlation coefficients of the ASD-MBQ and Asahide’s test. b Spearman’s correlation coefficients of the ASD-MBQ and SPQ. c Spearman’s correlation coefficients of the ASD-MBQ and PSI. (DOCX) [file pone.0267181.s002.docx]

**S2 Table a** Spearman's correlation coefficients of the ASD-MBQ and Asahide’s test

|  | Asahide’s test | | | | |
| --- | --- | --- | --- | --- | --- |
| ASD-MBQ | Communication skills | Daily living skills | Social skills | Interpersonal skills | Total score |
| Total Score | -.545** | -.608** | -.562** | -.595** | -.604** |
| Selective Eating | -.357** | -.371** | -.349** | -.381** | -.384** |
| Clumsiness/manners | -.646** | -.732** | -.666** | -.663** | -.704** |
| Interest in/concentration on Eating | -.305** | -.403** | -.368** | -.333** | -.369** |
| Oral-motor Function | -.178** | -.224** | -.180** | -.285** | -.230** |
| Overeating | -.411** | -.360** | -.386** | -.433** | -.419** |

**: P values showed < 0.001.

ASD-MBQ; Autism Spectrum Disorder-Mealtime Behavior Questionnaire Asahide’s test; Asahide’s test for social adjustment skills.

**S2 Table b** Spearman's correlation coefficients of the ASD-MBQ and SPQ

|  | SPQ | | | | | | | |
| --- | --- | --- | --- | --- | --- | --- | --- | --- |
| ASD-MBQ | tactile | taste and smell | Movement | Low response | Auditory | Low activity | Audio-visual | Total score |
| Total Score | .501** | .583** | .224** | .669** | .601** | .263** | .505** | .741** |
| Selective Eating | .487** | .766** | .212** | .341** | .390** | .195** | .424** | .583** |
| Clumsiness/manners | .380** | .403** | .222** | .649** | .510** | .185** | .405** | .616** |
| Interest in/concentration on Eating | .369** | .347** | .070 | .594** | .590** | .244** | .409** | .616** |
| Oral-motor Function | .286** | .232** | .159** | .494** | .367** | .196** | .302** | .457** |
| Overeating | .285** | .226** | .153** | .427** | .341** | .158** | .252** | .400** |

**: P values showed < 0.001

ASD-MBQ, Autism Spectrum Disorder-Mealtime Behavior Questionnaire; SPQ, the short Japanese version of the Sensory Profile.

**S2 Table c** Spearman's correlation coefficients of the ASD-MBQ and PSI

|  | PSI | | |
| --- | --- | --- | --- |
| ASD-MBQ | Recognition of negative emotions | Recognition of social activity limitation | Total score |
| Total Score | .496** | .300** | .455** |
| Selective Eating | .290** | .089 | .216** |
| Clumsiness/manners | .465** | .247** | .405** |
| Interest in/concentration on Eating | .401** | .365** | .435** |
| Oral-motor Function | .347** | .308** | .457** |
| Overeating | .310** | .189** | .280** |

**: P values showed < 0.001.

ASD-MBQ, Autism Spectrum Disorder-Mealtime Behavior Questionnaire; PSI, Parenting Strain Index.
