# Supplementary material for: Convergent validity of the Autism Spectrum Disorder Mealtime Behavior Questionnaire (ASD-MBQ) for children with autism spectrum disorder
Source: PLoS One. 2022 Apr 28;17(4):e0267181. doi: 10.1371/journal.pone.0267181 (PMC9049548; doi:10.1371/journal.pone.0267181)
Supplement: S3 Table — (DOCX) [file pone.0267181.s003.docx]

**S3 Table** the means and standard deviations of the age, ASD-MBQ, Asahide’s test, SPQ and PSI

n=294

ASD-MBQ, Autism Spectrum Disorder-Mealtime Behavior Questionnaire; Asahide’s test, Asahide’s test for social adjustment skills; SPQ, the short Japanese version of the Sensory Profile; SCQ, Social Communication Questionnaire; PSI, Parenting Strain Index
